# Aortoesophageal fistula as a late-onset complication of infected aortic arch aneurysm caused by *Salmonella enterica* serotype Choleraesuis: a case report

**DOI:** 10.1186/s12879-026-12654-7

**Published:** 2026-01-24

**Authors:** Hyo-Jin Lee, Yu-Min Han, Si-Hyun Kim

**Affiliations:** 1 Division of Infectious Diseases, Department of Internal Medicine, College of Medicine, The Catholic University of Korea, Seoul, Republic of Korea; 2 Vaccine Bio Research Institute, College of Medicine, The Catholic University of Korea, Seoul, Republic of Korea; 3 Division of Infectious Diseases, Department of Internal Medicine, Incheon St. Mary’s Hospital, College of Medicine, The Catholic University of Korea, 56, Dongsu-ro, Bupyeong-gu, Incheon, 21431 Republic of Korea

**Keywords:** Aortic aneurysm, Aortoesophageal fistula, *Salmonella enterica* serotype Choleraesuis

## Abstract

**Background:**

*Salmonella enterica* serotype Choleraesuis (*S.* Choleraesuis) is a nontyphoidal *Salmonella* serotype that can cause foodborne illness in a broad range of animals. In healthy individuals, *S.* Choleraesuis infections most often cause self-limiting acute gastroenteritis. However, more serious infections can develop and be associated with high mortality in immunocompromised patients.

**Case presentation:**

We report a 71-year-old male patient who presented to the emergency department with a 3-week history of fever and chest discomfort. Chest computed tomography revealed an infected aortic aneurysm in the distal aortic arch with an infected hematoma in the mediastinum. *S.* Choleraesuis was identified in both the blood cultures on admission and the hematoma specimen culture obtained from the surgical mediastinal drainage. Despite appropriate antimicrobial therapy with thoracic endovascular aortic repair and surgical mediastinal drainage, the patient died of massive hematemesis due to an aortoesophageal fistula.

**Conclusions:**

*S.* Choleraesuis tends to invade the bloodstream, leading to bacteremia and infected aortic aneurysms. Aortoesophageal fistula is a rare but life-threatening cause of upper gastrointestinal bleeding that may be due to an infected aortic aneurysm. To save the patient’s life, further studies on the proper management of infected aortic arch aneurysms complicated with aortoesophageal fistulas are needed.

**Clinical trial number:**

Not applicable.

**Supplementary Information:**

The online version contains supplementary material available at 10.1186/s12879-026-12654-7.

## Background

*Salmonella*, a genus of the family Enterobacteriaceae, spreads mainly by ingesting contaminated food or water or through contact with infected animals or persons. Nontyphoidal *Salmonella* (NTS) can colonize and infect the gastrointestinal tracts of a broad range of animals, including mammals, reptiles, birds, and insects, which serve as diverse reservoirs [1]. In healthy individuals, NTS infections most often cause self-limiting acute gastroenteritis. However, more serious infections, including bacteremia, metastatic infection, and endovascular infection, can develop and are associated with high mortality in immunocompromised patients [2]. This case report presents a patient with a late-onset aortoesophageal fistula secondary to an infected aortic arch aneurysm caused by *Salmonella enterica* serotype Choleraesuis (*S.* Choleraesuis) bacteremia.

## Case presentation

A 71-year-old male presented to the emergency room with a 3-week history of fever and chest discomfort. Despite a 7-day course of oral cefaclor, the fever persisted, and the patient’s chest discomfort gradually worsened. He had been on medication for hypertension and type 2 diabetes mellitus (DM).

**Fig. 1:**
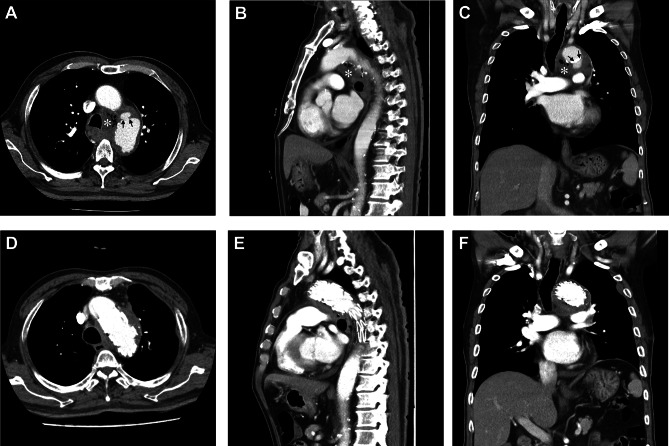
Axial, sagittal, and coronal views of contrast-enhanced chest computed tomography at admission (**A**-**C**) and on hospital day 49 (**D**-**F**). (**A-C**) Mediastinal fluid collection with mottled air density in the left lower paratracheal region (*asterisk*) and focal rupture in the distal portion of the aortic arch (*arrows*). (**D-F**) Improvement of the mediastinal abscess with favorable stent patency

On admission, his body temperature was 39.4 °C, while other vital signs remained stable. Physical examination revealed no other abnormalities. The laboratory findings were as follows: white blood cell count, 13,250/mm^3^ with 75.7% segment neutrophils (normal, 4,000–9,900/mm^3^ with 39–72% segment neutrophils); hemoglobin level, 13.6 g/dL (normal, 13.4–17.4 g/dL); platelet count, 255,000/mm^3^ (normal, 140,000–400,000/mm^3^); glucose level, 288 mg/dL (normal, 75–115 mg/dL); C-reactive protein level, 166.84 mg/L (normal, 0–5 mg/L); and HbA1c, 11.1% (normal, 4.3–6.3%). Contrast-enhanced chest computed tomography (CT) revealed focal luminal dilatation in the distal aortic arch and small fluid collection with mottled air density in the left lower paratracheal region, without clear evidence of contrast leakage (Fig. 1A-C). This suggested the possibility of an infected hematoma from focal rupture of the aortic arch or gas-forming mediastinitis. Esophagography and esophagogastroduodenoscopy (EGD) revealed no contrast leakage and no perforation, respectively. Additionally, bronchoscopy revealed no evidence of fistula in the trachea or bronchi.

Considering all these findings, treatment was initiated on the basis of the diagnosis of an infected hematoma due to focal rupture of an infected aortic arch aneurysm. Empirical antimicrobial therapy with ampicillin-sulbactam (3 g intravenously [IV] every 6 h) was administered, as this regimen provides broad coverage against both gram-positive and gram-negative organisms, including anaerobes, which are common pathogens in mediastinal and vascular infections. Thoracic endovascular aortic repair and surgical mediastinal drainage were performed on hospital days 2 and 4, respectively. Two days after admission, the ampicillin-sulbactam was replaced with piperacillin-tazobactam (4.5 g IV every 8 h) in response to preliminary blood culture results indicating the presence of gram-negative bacilli. The isolate was identified as *S. enterica* via conventional biochemical tests and the Vitek 2 system (bioMérieux, Marcy-l’Étoile, France). The serotyping was confirmed as *S.* Choleraesuis according to the Kauffman and White scheme (Becton Dickinson, Sparks, MD, U.S.A.). The results of antimicrobial susceptibility testing with the Vitek 2 system are summarized in Table 1. The bacterial culture of the infected hematoma specimen obtained during surgical mediastinal drainage again revealed *S.* Choleraesuis with the same antimicrobial susceptibility. The patient’s symptoms gradually improved, and the fever subsided on hospital day 5. All laboratory findings normalized except for a C-reactive protein level, which decreased to 9.50 mg/L on hospital day 7.

**Table 1:** Antimicrobial susceptibility testing results for *Salmonella enterica* serotype choleraesuis isolated from blood and hematoma specimen

Antimicrobial agents	MIC (mg/dL)	Interpretation
Ampicillin	≤ 8	S
Ampicillin/sulbactam	≤ 8/4	S
Aztreonam	≤ 4	S
Ciprofloxacin	≤ 1	S
Cefepime	≤ 2	S
Cefotaxime	≤ 1	S
Ceftazidime	≤ 1	S
Imipenem	≤ 1	S
Meropenem	≤ 1	S
Levofloxacin	≤ 2	S
Piperacillin	≤ 16	S
Piperacillin/tazobactam	≤ 16	S
Tigecycline	≤ 2	S
Trimethoprim/sulfamethoxazole	≤ 2/38	S

MIC = minimum inhibitory concentration, S = susceptible

**Fig. 2:**
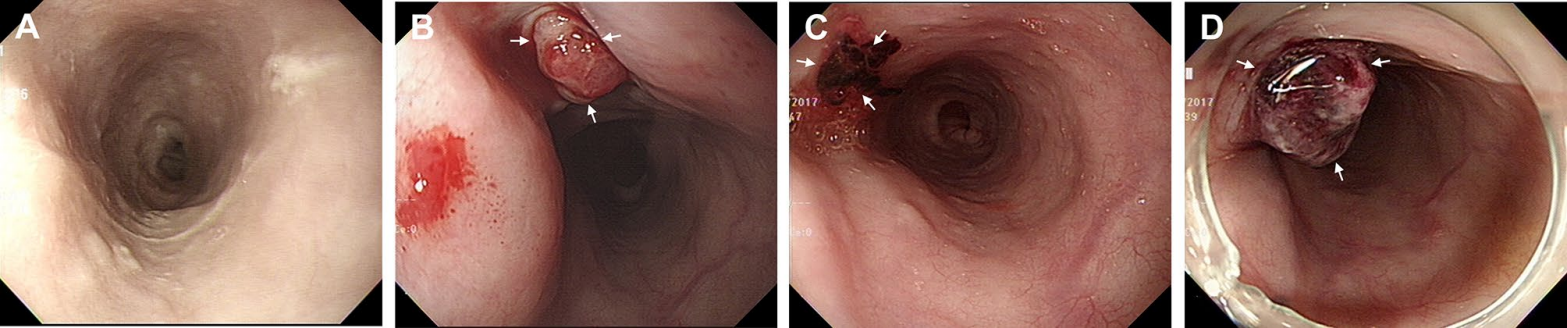
Esophagogastroduodenoscopy on hospital days 16 (**A**), 49 (**B**), 63 (**C**), and 67 (**D**). (**B-D**) Fistula opening with blood clots at the middle of the thoracic esophagus (*arrows*)

On hospital day 11, the patient developed dysphagia and new-onset fever. Follow-up chest CT demonstrated favorable stent patency without any evidence of endoleak; however, it revealed a residual organized hematoma in the mediastinum, which had marginally decreased in size but was still present. Despite appropriate antimicrobial therapy, the patient continued to complain of dysphagia, and fever persisted. Considering the superior tissue penetration and intracellular concentrations of fluoroquinolones, which is particularly advantageous in *Salmonella* infections, piperacillin-tazobactam was substituted with ciprofloxacin (400 mg IV every 12 h) on hospital day 13. Fever subsided from hospital day 16, but dysphagia persisted. Follow-up EGD revealed no abnormalities in the esophagus (Fig. 2A). Supportive care was continued with prolonged antimicrobial therapy with ciprofloxacin. Dysphagia gradually improved, and laboratory findings consistently remained within the normal range.

**Fig. 3:**
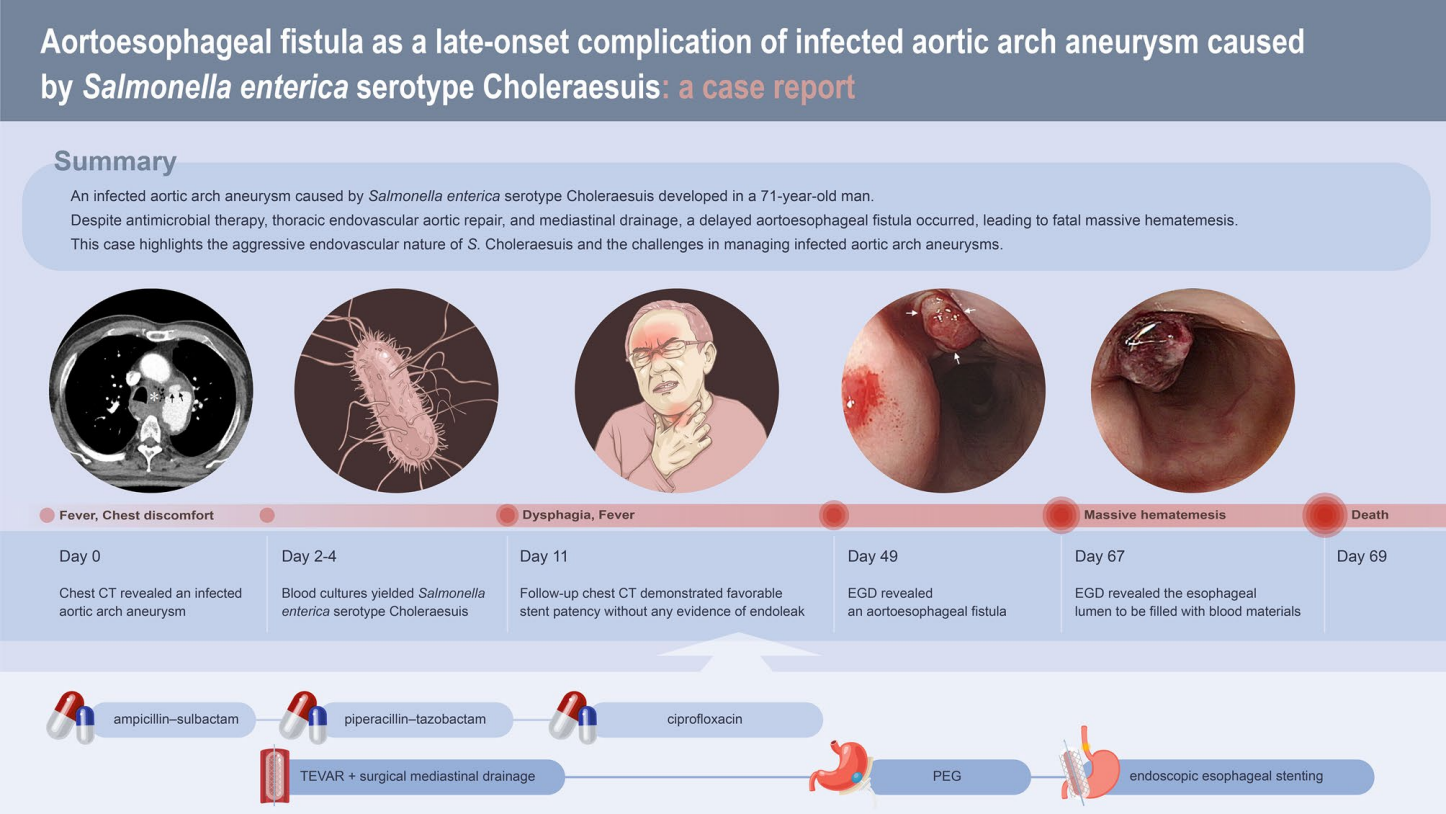
Graphical summary of the clinical course. CT = computed tomography, EGD = esophagogastroduodenoscopy, TEVAR = thoracic endovascular aortic repair

On hospital day 49, the patient complained of swallowing difficulty with solid food. Follow-up chest CT revealed further improvement of the mediastinal abscess (Fig. 1D-F). EGD revealed a fistula opening with blood clots at the middle thoracic esophagus, which was consistent with aortoesophageal fistula (Fig. 2B). The esophagogram revealed no evidence of contrast leakage or passage disturbance throughout the entire esophagus. The cardiothoracic surgeon recommended open graft explantation and repair of the esophagus, but the patient’s family did not consent to this surgery. As an alternative, percutaneous endoscopic gastrostomy was performed, and oral food intake was prohibited. The size of the fistula opening gradually decreased during the weekly follow-up EGD on hospital day 63 (Fig. 2C). The patient’s general condition also improved overall. However, on hospital day 67, sudden hematemesis occurred, and endoscopic stenting of the esophagus was performed as an emergency treatment (Fig. 2D). Despite endoscopic intervention and massive blood transfusion, the patient died of recurrent massive hematemesis on hospital day 69. A graphical summary illustrating the clinical timeline, diagnostic findings, and therapeutic interventions is provided in Fig. 3.

## Discussion and conclusions

The burden of *Salmonella* infection in humans and animals is significant worldwide. NTS infection typically results in self-limited acute gastroenteritis, but it can cause invasive infections associated with life-threatening complications, which depend on host factors and the specific *Salmonella* serotype [4, 5].

Enteric fever caused by typhoidal *Salmonella* strains, including *Salmonella enterica* serotypes Typhi, Paratyphi A, Paratyphi B, and Paratyphi C, has not been clearly associated with immunocompromised states. In contrast, NTS infection is known to be associated with a variety of immunocompromised conditions. Host risk factors for invasive NTS infection include extreme age, such as persons over 60 years of age and infants, advanced human immunodeficiency virus (HIV) infection, organ transplant recipients, corticosteroid or other immunosuppressive agents, malnutrition, and underlying chronic conditions such as sickle cell disease, chronic granulomatous disease, cirrhosis or cancer, atherosclerosis or prosthetic heart valves, and severe malarial anemia [2–5]. These factors increase susceptibility to severe infection and complications. In this patient, advanced age may have contributed to impaired host defense through age-related immunosenescence. His long-standing and poorly controlled DM is also a well-recognized risk factor for impaired host immunity. These factors together likely predisposed the patient to invasive NTS infection despite the absence of HIV infection or other overt causes of immunosuppression.

Additionally, serotypes of NTS are correlated with specific clinical features and complications. *S.* Enteritidis and *S.* Typhimurium are the most common causes of invasive NTS infection. *S.* Choleraesuis, an infrequent NTS serotype, is highly pathogenic to humans and is more likely than other NTS serotypes to cause bacteremia and endovascular infection in humans [6–8]. Endovascular infection due to NTS is associated with atherosclerosis, without the presence of other clinical features or immunocompromised conditions [9, 10]. Individuals of advanced age, as in our case, are more likely to develop diabetes mellitus and hypertension, with atherosclerosis also being a common comorbidity. The NTS organisms have a propensity to invade preexisting atherosclerotic sites of large vessels and cause endothelial infection. A previous study reported that the incidence of endovascular infection was 18.5% in elderly patients aged 55 years and older, whereas it was 2.7% in young patients [11].

Infected aortic aneurysm, an endovascular infection, is an uncommon but serious complication of NTS infection that is associated with significant morbidity and mortality. *Staphylococcus aureus* is one of the most common causative pathogens of infected aortic aneurysms, typically inducing vascular injury through acute suppurative inflammation following contiguous spread or septic embolization. By contrast, *Salmonella* species are facultative intracellular pathogens that disseminate hematogenously and preferentially localize to atherosclerotic or damaged arterial walls. This difference in pathogenesis accounts for the rapid expansion, higher risk of rupture, and higher mortality rate often observed in *Salmonella*-associated infected aortic aneurysms despite appropriate therapy. The abdominal aorta, especially the infrarenal aorta, is the most frequent location of infected aortic aneurysms caused by *Salmonella* spp., while the involvement of the thoracic aorta and aortic arch is rare [12–14]. Table 2 summarizes recently reported cases of *Salmonella*-associated infected aortic aneurysms [13–19]. A recent systematic review on contemporary outcomes of endovascular repair and open surgical repair for the treatment of infected aortic aneurysms was performed [20]. The authors suggested that endovascular repair is as effective and safe as open surgical repair for the descending thoracic and abdominal aorta. However, infected aortic arch aneurysms accounted for only 2.2% of all cases, resulting in insufficiently powered evidence to support endovascular repair. Moreover, infected aortic arch aneurysms have an increased rate of rupture because of increased blood pressure and shear stress. However, endovascular repair can be considered a less invasive alternative and an emergency intervention to achieve immediate bleeding control and stabilize the patient’s condition when open surgery is not feasible due to extremely high operative risk [21].

**Table 2:** Summary of recently reported cases (2015-present) of infected aortic aneurysm caused by *Salmonella* species

Author (Year)	Age/Sex	Organism	Aortic Site	Presence of AEF	Intervention	Outcome
Pasveer et al. (2017) [15]	58 / M	*Salmonella enterica* serotype Enteritidis	Abdominal aorta, iliac artery	No	Open surgery	Survived
Aftab et al. (2019) [16]	88 / M	*Salmonella* spp. (species not specified)	Aortic arch	No	TEVAR	Died
Akishima et al. (2020) [14]	66 / M	*Salmonella* spp. (species not specified)	Aortic arch	Yes	Open surgery + TEVAR	Survived
Rumoroso et al. (2022) [17]	78 / M	*Salmonella* spp. (species not specified)	Infrarenal abdominal aorta	No	Open surgery	Survived
Kim et al. (2023) [13]	66 / M	*Salmonella* spp. (species not specified)	Aortic arch	No	None	Survived
Wahab et al. (2023) [18]	54 / M	*Salmonella enterica* serotype Enteritidis	Infrarenal abdominal aorta	No	Open surgery	Survived
Yoshida et al. (2023) [19]	62 / M	*Salmonella* spp. (species not specified)	Ascending aorta, aortic arch	No	Open surgery	Survived

AEF = aortoenteric fistula, TEVAR = thoracic endovascular aortic repair

Aortoesophageal fistula, a complication of infected aortic aneurysm rupture, is very rare, and its treatment remains challenging [22–25]. It causes massive upper gastrointestinal bleeding and hypovolemic shock and is always fatal if left untreated. Early diagnosis and aggressive surgical treatment are the best ways to achieve optimal outcomes. However, unfortunately, most patients with this condition are elderly with multiple comorbidities and poorer medical status, posing substantial obstacles for surgical intervention, as demonstrated in this case.

Aortoesophageal fistula developed in this patient as a late-onset complication of infected aortic arch aneurysm caused by *S.* Choleraesuis. Several mechanisms may have contributed: (1) persistent localized infection and chronic inflammation leading to progressive weakening of the aortic and esophageal walls, (2) persistent tissue damage caused by the residual mediastinal hematoma, and (3) gradual erosion of the infected aortic aneurysm into the adjacent esophagus despite initial clinical improvement. The limited number of reported cases has led to a lack of experience and knowledge in treating this condition. Despite our best efforts to treat the patient, the outcome was fatal. However, this case report provides valuable information for understanding this condition and contributes to the investigation of optimal treatments.

In summary, this case highlights the challenges in managing infected aortic aneurysm and its devastating complication of aorto-esophageal fistula caused by *S.* Choleraesuis. Although endovascular treatment allowed temporary stabilization, the outcome remained poor in the absence of definitive surgical repair. Furthermore, because NST infection is not classified as a legally notifiable infectious disease in our country, we were unable to perform a comprehensive epidemiological investigation, and this should be acknowledged as a limitation of this case report.

## Supplementary Information

Below is the link to the electronic supplementary material.

Supplementary Material 1

## Data Availability

The data used during the current study are available from the corresponding author upon reasonable request.

## Abbreviations

NTS: Nontyphoidal *Salmonella*
S. Choleraesuis: *Salmonella enterica* serotype Choleraesuis
CT: Computed tomography
EGD: Esophagogastroduodenoscopy
IV: Intravenously
HIV: Human immunodeficiency virus
