# Supplementary material for: Aortoesophageal fistula as a late-onset complication of infected aortic arch aneurysm caused by Salmonella enterica serotype Choleraesuis: a case report
Source: BMC Infect Dis. 2026 Jan 24;26:388. doi: 10.1186/s12879-026-12654-7 (PMC12911385; doi:10.1186/s12879-026-12654-7)
Supplement: Supplementary file 1 — Supplementary Material 1 [file 12879_2026_12654_MOESM1_ESM.docx]

| **Item** | **Description** | **Location in Manuscript** |
| --- | --- | --- |
| 1. Title | Identification as a case report | Title page |
| 2. Keywords | Three key terms included | Abstract |
| 3. Abstract | Structured summary provided | Abstract |
| 4. Introduction | Background and rationale | Background |
| 5. Patient Information | Demographics, medical history | Case presentation |
| 6. Clinical Findings | Physical exam & symptoms | Case presentation |
| 7. Timeline | Sequential clinical course | Figure 3 |
| 8. Diagnostic Assessment | Labs, imaging, differential | Case presentation |
| 9. Therapeutic Intervention | Antimicrobial therapy, TEVAR, surgical drainage, endoscopic stenting | Case presentation |
| 10. Follow-up & Outcomes | Clinical progression, complications, final outcome | Case presentation |
| 11. Discussion | Interpretation, comparison with literature | Discussion and conclusions |
| 12. Patient Perspective | Not available | Not applicable |
| 13. Informed Consent | Written consent obtained | Stated in Declarations |

**CARE Checklist – Case Report**
